# Cosmetic patent and female invention

**DOI:** 10.1371/journal.pone.0305238

**Published:** 2024-08-08

**Authors:** Jong Wook Lee, Eunji Jeon, So Young Sohn

**Affiliations:** Dept. of Industrial Engineering, Yonsei University, Seoul, Republic of Korea; National Taiwan University, TAIWAN

## Abstract

Majority customers of cosmetics are female. Would this imply a high proportion of inventors of cosmetics technology is female? Would the inventor’s gender be related to the characteristics and quality of corresponding patent? This study tries to identify manifestation of gender equity in cosmetics technology in terms of patent application and grant, technical characteristics, and its performance. We apply topic modeling, zero-inflated Poisson regression, and survival analysis to patents related to cosmetics that were applied to the United States Patent and Trademark Office from 1970 to 2016. The results show that women’s participation in cosmetic inventions is becoming active and has experienced many changes in technical characteristics, but in terms of performance, it is still sluggish. This study is expected to contribute to deepening our understanding about gender issues in technology development.

## Introduction

Cosmetic technology was born in Egypt 12,000 years ago and has a very long history. In the past, the majority demand for cosmetic technology was from adult female users, but recently its market has been increasing regardless of age or gender. Not only the socio-demographic market for customers, but also the cosmetic technology area has been extended to that of pharmaceutical and medicine. As cosmetic technologies become very closely related to human life in various ways, cosmetic research have been actively conducted and various inventions have been granted in the form of patent. Accordingly, inventors with various backgrounds are involved in cosmetics patenting, which can affect the patent characteristics and quality. However, not much effort has been put to analyze what factors are associated with the characteristics and quality of cosmetics patents.

In the past, women’s participation in the fields of science, technology, engineering, and mathematics (STEM) has been extremely limited [1]. But today, gender gaps in educational opportunities have been decreased significantly, and women are increasingly participating in STEM fields [2]. Subsequently, the number of female inventors has been increasing and studies on the changes of women participation in science and technology fields are gaining lot of attention. Studies have pointed out the differences between male-and female-dominated STEM fields [3] and the technological fields of interest. Particularly, the female-majority teams are more likely to invent technologies for women, especially in the health and chemistry areas [4, 5]. Meanwhile, the project SAPPHO, which studied differences between success and failure in innovation also concludes that successful innovators have better ability to understand user needs correctly than failed innovators [6, 7]. As the majority of customers of cosmetic inventions are women, female inventors are more likely to be in position to appreciate user needs. Thus, in the field of cosmetic technology, examining the participation of women is necessary for a deeper understanding of the characteristics and quality of cosmetics patents.

Prior studies have suggested that the characteristics and the performance of the patents invented by females may differ from those invented by males, such as in their applications and grants, citation counts, and technological fields [4–8]. However, the differences in technological attributes and performance by female participation has been under-researched in cosmetics technology. The investigation of the patenting activities of female and the characteristics of their patents may provide a comprehensive understanding of status quo of female participation in developing cosmetic technology. Therefore, this study aims to identify manifestation of gender composition of inventor teams in cosmetic technology. Patent data is used as an indicator of cosmetic technology and the inventor’s gender is used to determine the contribution of female participants in the patent.

The purpose of this study is to identify the contribution of women inventors in cosmetic patents in terms of the following three research questions:

1. Would the female participation in invention be positively related to the number of patent applications, and their grant rate and grant lag?
2. Is there significant association between female participation in invention and the technological characteristics of cosmetic patents?
3. Do the cosmetics patents invented by female have high performances compared the rest of the others?

By answering these questions, this study illustrates the differences of patenting trends by the participation of women inventors in cosmetics from various perspectives. Our findings propose that there exists increasing participation of female inventors which brought many changes in characteristics of cosmetic technology as in various different sectors [4–8], yet the performance of the cosmetic patents invented by females still lags behind. The result of our analysis can be used to back up R&D initiatives in terms of R&D strategy and manpower plan for team formation by taking advantage of gender characteristics in developing cosmetics technology. Moreover, this study contributes to the literature dealing with gender disparities in patenting activities in one of the more balanced fields with less gender gap. It can enhance the understanding of gender association with characteristics of developed technology and its performance.

The rest of this paper is organized as follows. In Section 2, we review the previous studies on the gender research in invention. In Section 3, we introduce the patent data and preprocessing process. In Section 4, we compare the characteristics of cosmetics patents that depend on the participation of women. Finally, in Section 5, we present the conclusions as well as the contributions of this study.

## Literature review

### Female engagement in STEM

Until the early 2000s, there was a culture in which women’s participation in the STEM sector was limited and socially reluctant. However, as perceptions of gender equality spread, women’s entry into the STEM fields has been increasing as the gap of their status and education opportunities to those of men is decreasing [9].

Conversely, gender segregation depending on the field of science discipline has become an issue. Recent research confirms the existence of varying levels of gender segregation in distinct fields of study ultimately disadvantaging women obtaining authority positions [10, 11]. Insights into the gender hierarchy within professions of academic science can be gained by understanding how scientists perceive different degrees of gender segregation among various fields of science. Prior research confirms the prevalence of the perceived gender role stereotypes in research disciplines in traditionally male-dominant businesses [12].

Mentors and role models discreetly dissuade a woman from sticking with a particular discipline if scientists themselves believe that one is more difficult for women to succeed in than another, which influences women’s thinking as they enter a particular science discipline [13]. Thus, many women may be drawn to the feminizing field of cosmetic science. Therefore, relatively active female participation can be expected in such research domains.

### Women’s contribution in patenting activity

The trend of growing participation of females has been observed not only in the education or professions in STEM but also in the filing activities of intellectual property rights. In patenting activities, women’s contributions have been increasing in most countries in both the United States Patent and Trademark Office (USPTO) and the Patent Cooperation Treaty (PCT) applications [14, 15]. Specifically, Frietsch et al. [16] suggested that women’s contributions have increased in all countries between 1991 and 2005. In particular, women’s contribution in Spain was highest between 2003 and 2005. During 2011–2015, China, South Korea and Singapore accounted for the highest proportion of women’s participation in patent inventions, while Germany, Japan and the United Kingdom accounted for the poorest countries [15]. However, still, the patents applied at the USPTO and PCT during late 1990s to early 2010s had shown that the patent application acceptance of female was lower than that of male applicant [8, 14, 15, 17, 18]. In addition, the grant rate of female patent was much lower [8, 17] and approved number of claims was also smaller than that of male patents [8].

Recently, women are increasingly likely to patent in a large, gender-mixed inventor teams [17], highlighting the growing importance of exploration of innovative mixed gender collaboration. In particular, the trend of biotechnology has been shifting from invention by one gender to co-invention [19]. In addition, among the type of the assignee, such as university, company, and individual, patents from university had the highest female participation [14]. The proportions of female inventorships in university are relatively superior across all macro technological areas, with the exception of areas such as games, furniture, and other consumer goods [14]. Hence, investigating the changes in women’s participation in technology development may provide an understanding of the patenting trends of women inventors and gender disparities of patents.

### Differences in patenting fields by gender

There exist some differences on patenting activities by gender. Among various technological fields, women inventors turn out to be largely focused on life sciences fields, such as biotechnology, pharmaceuticals, and agriculture [20]. On the contrary, several fields, such as engineering, physical sciences, mathematics, and transport, have low share of female inventions [21]. Specifically, Frietsch et al. [16] addressed that the women’s contribution had largest increase from 1991 to 2005 in chemistry field among the five large technical areas (Chemistry, Electrical, Instruments, Mechanical, Other). Similarly, among the patents filed in 2010, the proportion of patents including female inventors was the highest at 50% in technology class “Chemistry: Natural Resins or Derivatives” [4]. Subsequently, “Organic Compounds Drug”, and “Bio-Affecting and Body Treating Compositions” accounted for a high percentage. Meanwhile, in 2015, biotechnology (BT) (57.6%), pharmaceuticals (55.5%) and organic chemistry (50.7%) dominate other fields of technology on which women inventors participated, whereas the machinery sector is recorded to have lowest share among the fields [15]. This suggests that there exist the differences in the participation of female inventors by technological sectors.

As such, the female-majority teams are more likely to invent technologies for women, especially in the health and chemistry areas [4, 5]. Considering the much greater number of male inventors in overall patents, the technologies related to women’s health can be represented as more balanced fields in terms of the number of patents than other fields, overcoming the difference in the total number of inventions between women and men. Especially, the cosmetics industry was traditionally focused on women, which can be expected to be one of the gender-balanced technological field. These gender-balanced fields may allow fair comparison of technological trends or innovation performance by gender.

Recently, cosmetics is no longer a preserve of women, which is shown by the increasing spendings on cosmetics of male [22]. The gender differences lead to different motivations of using cosmetics and consumption behaviors. However, existing studies on cosmetics lack the exploration of innovation characteristics and performance by gender diversity of inventor groups. Hence, research in cosmetics can provide insightful implications on the gender differences and the synergistic effects of gender diversity in patenting activities.

## Data and pre-processing

The purpose of this study is to identify the gender implications of cosmetic technology development. We wanted to identify female participation in cosmetic technology development and diffusion of cosmetic technology invented by women. So, we conduct empirical studies based on patent data, which provide a dependable information about the technologies [23].

**Fig 1:**
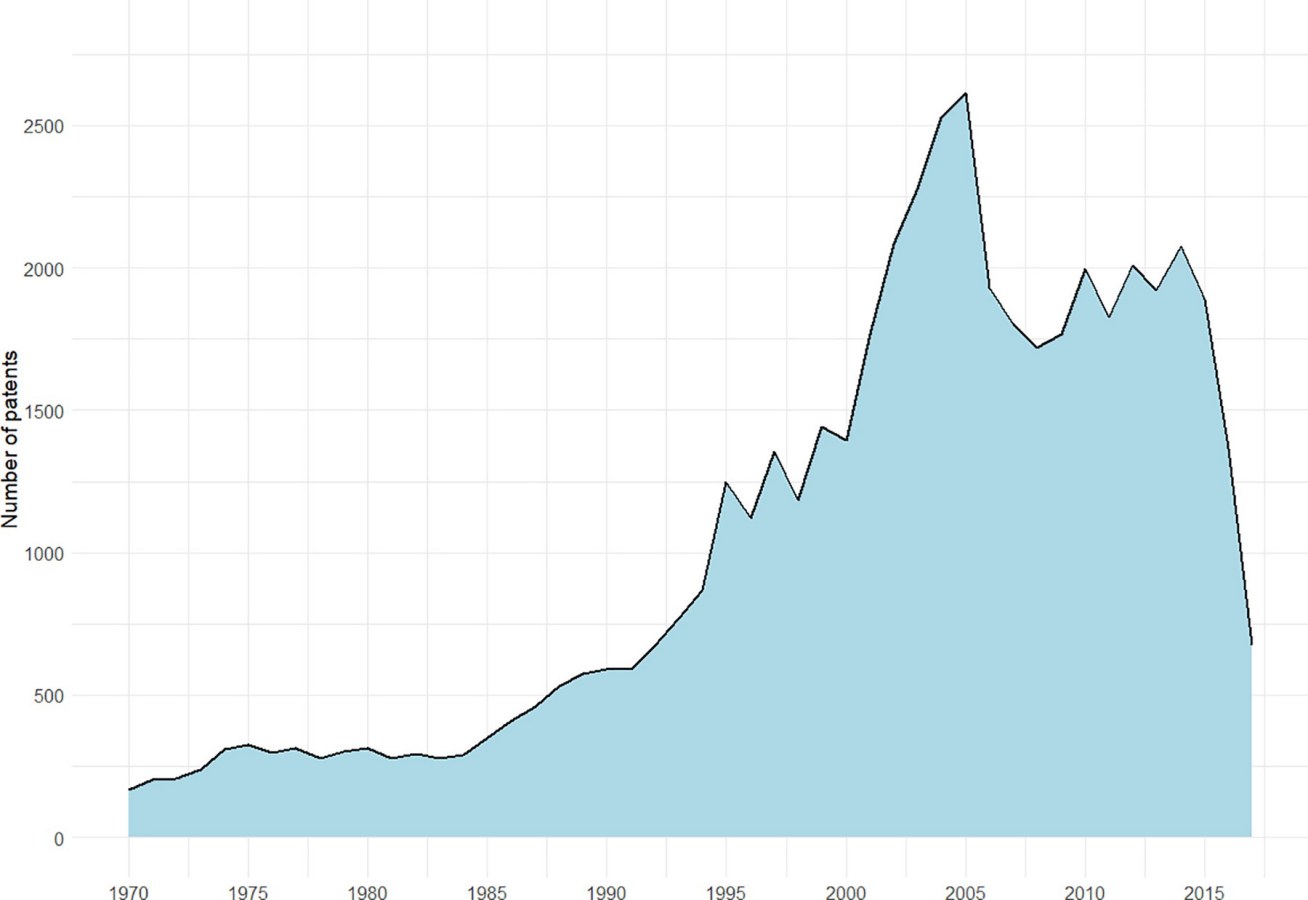
Change in the number of cosmetic technology patents applied.

First, we collected the patents related to cosmetics that were applied to the USPTO from 1970 to 2016 using the PATSTAT database. The cosmetic-related patents were retrieved based on International Patent Classification (IPC) code, which represents the technical area covered by patents: A61Q (Specific use of cosmetics or similar toilet preparations) and A61K 08 (Cosmetics or similar toilet preparations). As a result, 49,241 patents are collected, and their annual application trend is shown in Fig 1. The application number of cosmetic technology showed a big rise since 1984 and peaked in 2005. Since then, it has stabilized in a slightly reduced state and has stalled in 2016.

However, there is a difficulty in obtaining information about the gender of the patent inventor. Bibliographic information in a patent includes the inventor’s name, address, and nationality, but no gender information. Since the patent data do not explicitly provide gender information for inventor, many previous studies have suggested to find gender information through the names of patent inventors. Existing studies have created gender dictionaries by inventors’ name and indexed their gender to analyze the innovation activities by gender. Thus, we used three well-known databases to distinguish the gender of the inventor. The first is *the Gender profiles in worldwide patenting* database provided by the UK Intellectual Patent Office. The 2019 version contains more than 30 million gender information. This database also has the advantage of being compatible with PATSTAT. We identified the gender of 57,805 out of 67,211 inventors of the 49,241 cosmetic patents. In addition, we used the World Gender-Name Dictionary (WGND) provided by World Intellectual Property Organization (WIPO) as the second database to index the gender of the remaining 9,406 inventors. The WGND contains 6,247,039 unique pairs of names and countries. We could find additional genders of 5,735 patent inventors. Finally, we used the baby names with their genders provided by The United States Social Security Administration. We could find additional genders of 163 patent inventors. In sum, we were able to assign the gender of 63,703 inventors out of 67,211 inventors.

**Fig 2:**
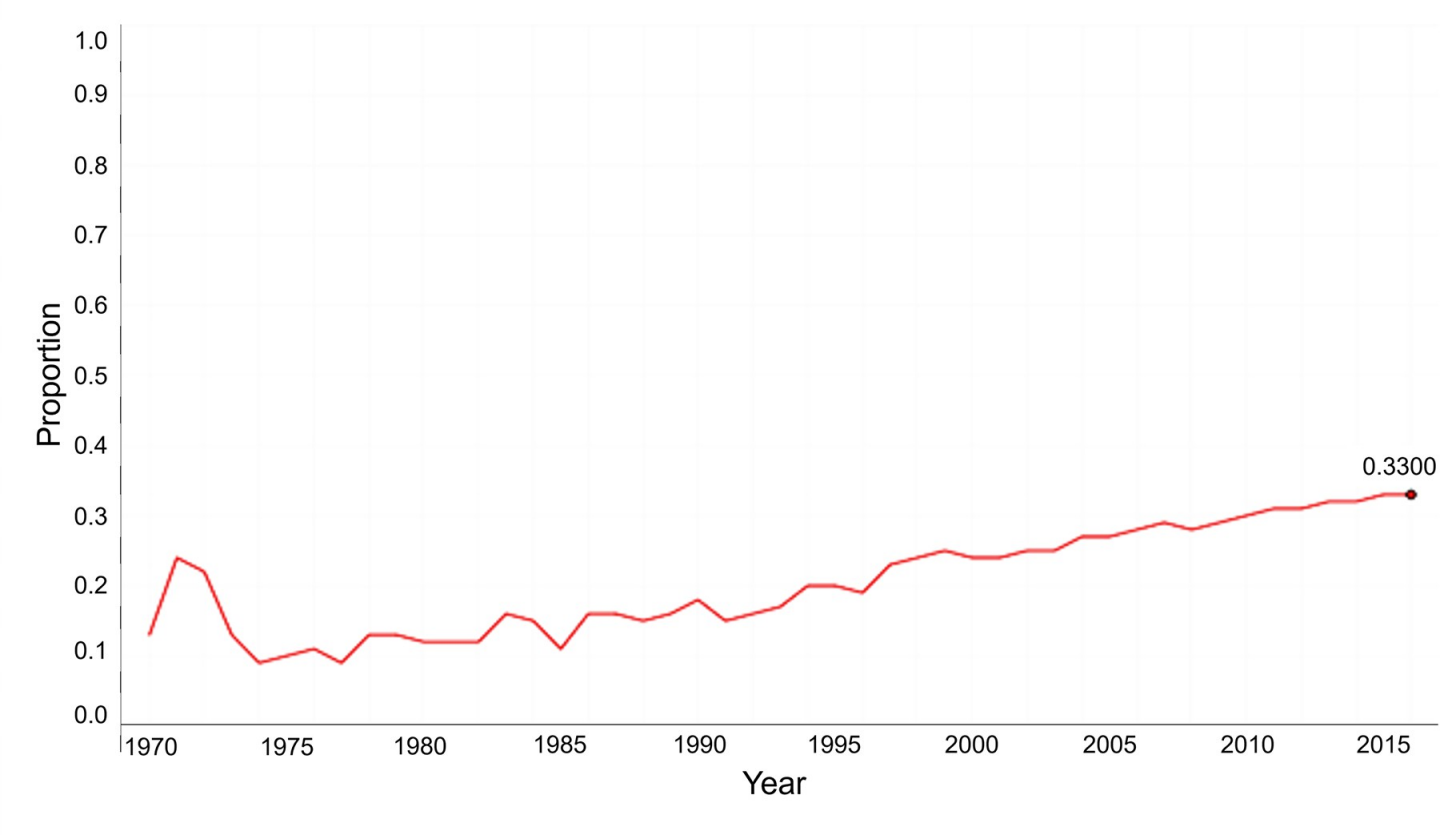
Changes in the proportion of cosmetic patents with the participation of women inventors.

Fig 2 shows the change in the proportion of women inventors of cosmetics patents from 1970 to 2016. In 1970, the ratio of women was very low at 12.28%, but it gradually increased with time resulting in 33.72% in 2016. In the development of cosmetic technology, the participation of women has been spreading more and more.

Then, we define the gender attribute of a patent, which can consist of more than one inventor. There are three conventional approaches proposed to represent a gender implication of a patent. The first way is to use the proportion of women among the inventors of the patent [8, 14, 16]. The second way of representing the female contribution of patent is by classifying patents into three cases, that are whether the inventors of the patent are all female, all male, or mixed genders [18, 19]. The third way is to check whether if there is at least one female among the inventors [4, 15, 17]. In this study, our primary objective is to investigate the contribution of female inventors in cosmetic patents, comparing the patenting patterns and disparities between different gender groups. Hence, we classify the gender implications of cosmetic technology into three cases: (1) the inventors of the patent are all females, (2) all males, and (3) mixed genders [19, 24]. We removed 1,857 patents out of 49,241 patents which we cannot identify the gender of the majority inventors. As a result of removing them from the count, patents of male inventors, female inventors, and mixed gender are classified as 25,123, 3,937, 18,324, respectively.

## Empirical analysis

### Female participation in cosmetics patent application / granted

**Fig 3:**
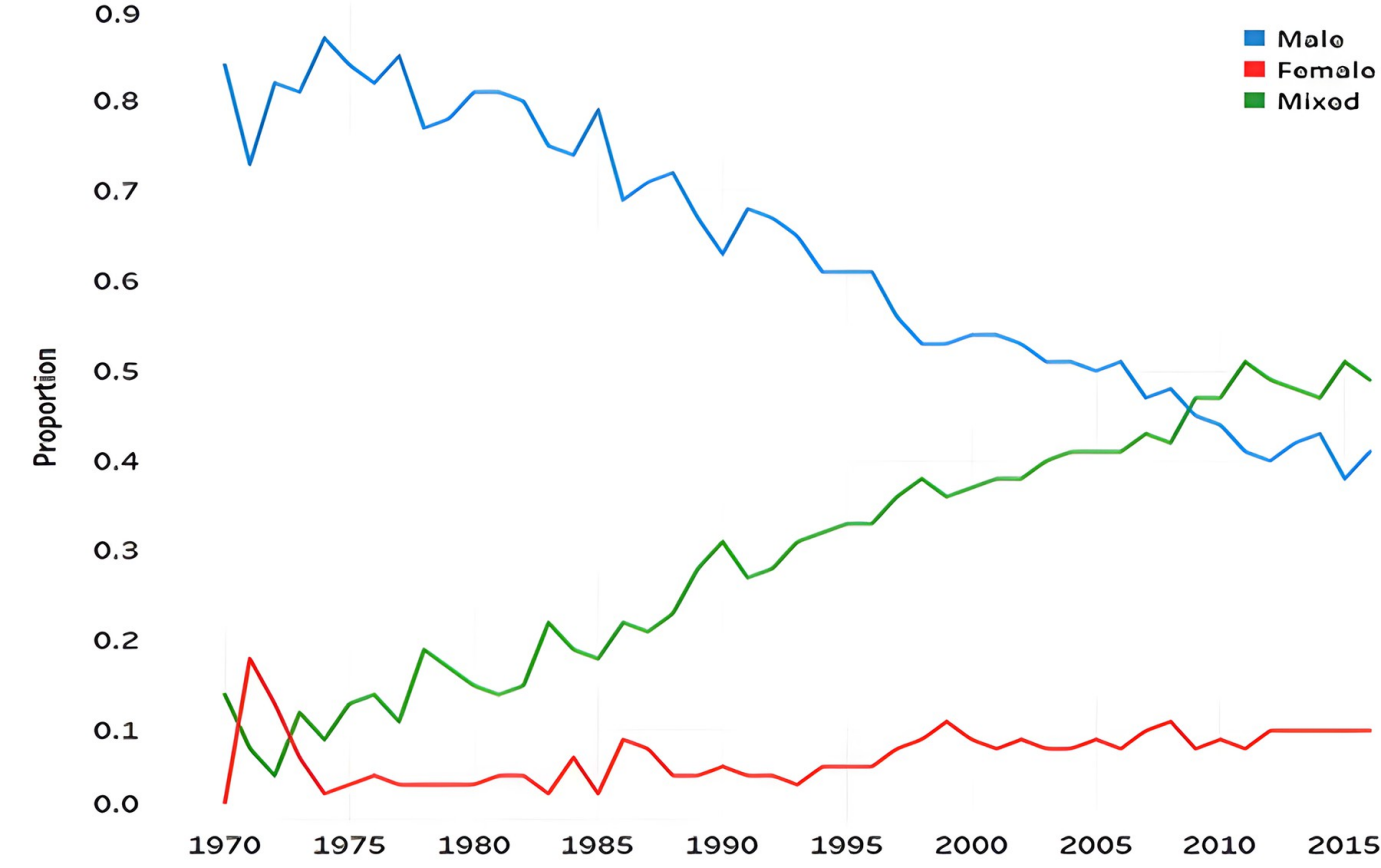
Annual change in cosmetics patent application rate by the gender of patent inventors.

Fig 3 shows the annual change in patent application rate by gender of cosmetics patent inventor. Until the early 1990s, most of the patents were applied only by male inventors. However, since then, the proportion of mixed patents has risen sharply, and in 2009 it overwhelmed patents with only male inventors. The proportion of patents with female inventors have also risen steadily and have remained at 10% since 2012.

**Fig 4:**
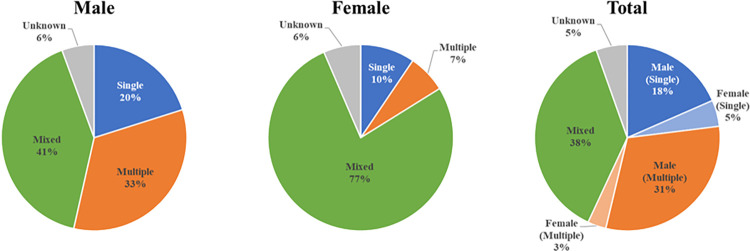
Proportion of patent application by the gender of patent inventors.

Fig 4 shows the proportion of patent application by gender of inventors during 1970–2016. The patents invented by a group of mixed gender had the highest proportion of patent applications compared to those invented by single gender groups. Within a single gender group of inventors, women tend to invent alone rather than collaborators compared to men. Patents invented by single female inventor was 10% of the total patents with participation of female inventors (which exceeds that of multiple female inventors), whereas the proportion of patent applications with a single male inventor is lower than that of multiple male inventors. When calculating the average number of inventors of at least two inventors, a patent with male only has a value of 2.93, a patent with female only of 2.38, and a mixed patent of 3.89. These indicate that the patents invented by female-only groups are less likely to be associated with collaborative activities and tend to be usually developed by sole inventors. On the other hand, in mixed patents where women and men collaborate, cooperation is occurred more as a larger team.

Table 1 shows the difference in patent grant according to the gender of the patent inventor. Grant lag refers to the period of time between the filing date of patent application and the date of its grant, which is shown to have inverse relation with patent value [25]. The table shows the highest rate of grant assigned to patents developed by male. On the other hand, the rate of grant of patents by female was the smallest. The chi-squared test indicates there are significant differences on the distribution of a categorical variable, that is, if patent is granted or not, between the inventor groups. Overall, the grant rate has significant differences by group, and decreases with the participation of women in patent inventions. We also observed differences in grant lag by gender for granted patents. The grant lag of a patent is influenced by the nature of the patent and generally averages 28 months [26]. In contrast, the average grant lag of cosmetic technology patents is observed at 35 months. The average grant lag of male, female, mixed was 32.62, 35.05, and 37.21, respectively. The analysis of variance (ANOVA) test showed at least one pair of groups has statistically significant difference. Then, we conduct post-hoc test, specifically the Games Howell test, considering that the size of sample is different, and the homogeneity of variance is not satisfied, which is proven by Levene test [27]. The post-hoc test showed every pair of groups had statistically significant differences. The inventions with the female-invented patents had significantly longer grant lag than male inventions. Surprisingly, it took the longest time for mixed patents to be granted. Overall, women’s inventions not only had a low grant rate, but also took a long time to be granted than men’s inventions.

**Table 1:** Comparison of grant information by patent gender.

Indicator	Gender			Statistical significance
	Male	Female	Mixed	
**Proportion of granted patents**	70.9%	56.4%	60.4%	Chi-squared test ***
**Average grant lag**	32.62	35.05	37.21	ANOVA ***
				Levene ***
				Games Howell ***

Significance: <0.001: ‘***’, <0.01: ‘**’, <0.05: ‘*’, >0.05: ‘-’

### Technological characteristics by gender

We observed how bibliographic information, which stands out the technical characteristics of patents, differs by gender. First, claims, backward citation, and family size were chosen as comparison factors. The claims in a patent have separate legal effects and expresses multiple technical features within an invention, which indicate the inventiveness and the scope of protection of patents [28, 29]. Tong and Frame [30] insisted that the number of patent claims is a better indicator of technological performance than just patent counts. As the patent fee generally relies on the number of claims, the claim counts reflect the financial value of the invention [31–33]. Backward citation notes the earlier patents that a given patent cited [28, 34–36]. Inventions containing fewer backward citations indicate higher level of novelty in their features or functionalities (i.e., novelty in application), suggesting previous inventions have less bearing on the invention [37]. A patent family is “the same invention disclosed by a common inventor and patented in more than one country.” The family size represents the number of jurisdictions that the patent has been sought for, which indicates the solid legal protection of patent rights recognized by several countries [25, 29, 30]. Hence, the patent family was used as an indicator to evaluate the market value of patents in various studies [34, 38].

**Fig 5:**
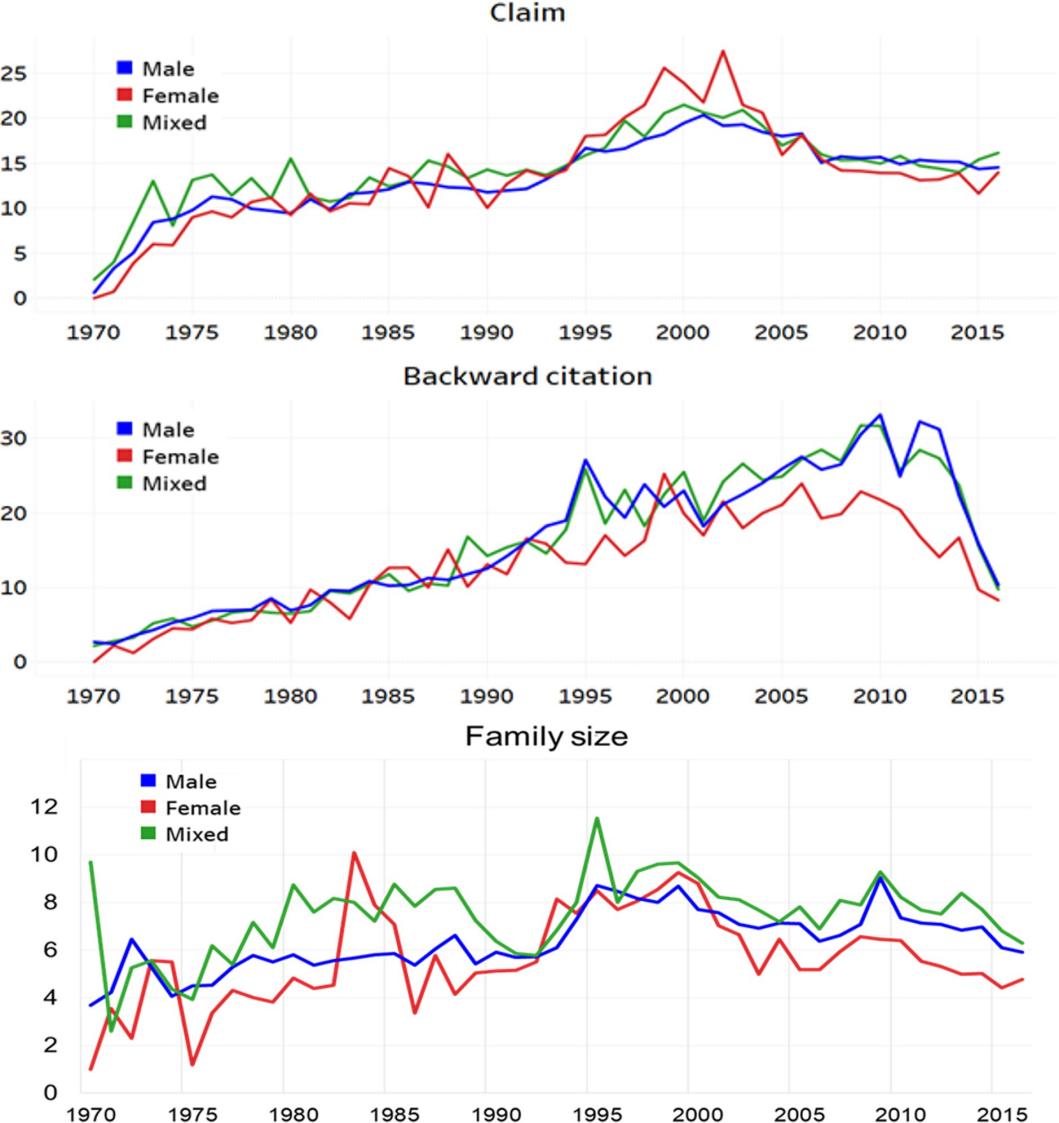
The annual trend of patent claims (top), backward citations (middle) and family size (bottom) by gender.

Table 2 shows the average of three factors by patent inventor gender, and test results for significance for their differences. Surprisingly, female participation showed a statistically significant positive correlation with the number of claims. However, the difference between patents with female and mixed inventors was not statistically significant. Top graph of Fig 5 shows differences in the number of claims by gender between 1970 and 2016. The trend in claim numbers is similar regardless of gender. Between 1995 and 2003, the number of claims for female was the highest, but it has been the least since then. In most years, patents with female participation have many claims rather than patents whose inventors are only males. For the remaining two factors, patents of male dominated female inventors. In addition, two factors are higher when male and female inventors interact compared to when the inventor of the patent is biased by one gender. The graph in the middle of Fig 5 shows the change in the citation counts over time by gender. The backward citations showed the same pattern regardless of gender, and in most of the time, female has less backward citations than other groups. The less backward citations indicate the power to voluntarily create novel inventions in a positive sense [40]. Lastly, the bottom graph shows the differences in the family size by gender. In most of the time, the patents of an inventor group with mixed gender had greatest family size than those of the single gender groups. Moreover, the patents invented by male-only group had larger number of family patents compared to those of female-only group.

**Table 2:** Comparison of bibliometric information by the gender of patent inventors.

Indicator	Gender			Statistical significance		
	Male	Female	Mixed	ANOVA	Levene	Games Howell
**Claim**	15.05	16.96	16.69	***	***	Female-Mixed (-)
						Female-Male /
						Male-Mixed (***)
**Backward citation**	20.77	17.29	23.2	***	***	***
**Family size**	6.03	5.52	6.85	***	*	***

Significance: <0.001: ‘***’, <0.01: ‘**’, <0.05: ‘*’, >0.05: ‘-’

Second, we observed how the technical areas covered by patents differed by gender. We used six-digit IPC codes that can specify and differentiate cosmetic technology. Table 3 shows top ten IPC codes for male, female, and mixed inventors. The ratio of the occurrence of IPC codes in total patents in cosmetics by gender indicates the degree of the technical area accounting for cosmetics technology. Regardless of the gender of the patent inventors, techniques for skin and hair care accounted for a high proportion and were connected to medical technology. For patent inventions with women participation, interest in makeup-related technologies increases greatly, but interest in dental care-related technologies decreases significantly.

**Table 3:** Top 10 six-digit IPC codes by gender.

IPC	Description	Rank by gender (ratio)		
		Male	Female	Mixed
**A61K 6**	Preparations for dentistry	1 (0.98)	-	-
**A61K 8**	Cosmetics characterized by compositions including oils, organic and inorganic ingredients	-	1 (0.978)	1 (0.981)
**A61Q 19**	Preparations for care of the skin	2 (0.386)	2 (0.429)	2 (0.428)
**A61Q 5**	Preparations for care of the hair	3 (0.272)	3 (0.332)	3 (0.324)
**A61K 31**	Medicinal preparations containing organic active ingredients	4 (0.206)	6 (0.157)	4 (0.200)
**A61Q 11**	Preparations for care of the teeth, of the oral cavity or of dentures	5 (0.171)	10 (0.069)	8 (0.122)
**A61Q 17**	Barrier preparations; Preparations brought into direct contact with the skin for affording protection against external influences	6 (0.161)	5 (0.158)	5 (0.172)
**A61K 9**	Medicinal preparations characterized by special physical form	7 (0.148)	7 (0.102)	6 (0.133)
**A61K 47**	Medicinal preparations characterized by the non-active ingredients	8 (0.100)	-	10 (0.086)
**A61K 36**	Medicinal preparations of undetermined constitution containing material from algae, lichens, fungi or plants, or derivatives thereof	-	8 (0.091)	-
**A61Q 1**	Make-up preparations; Body powders; removing make-up	9 (0.096)	4 (0.200)	7 (0.131)
**A61P 17**	Drugs for dermatological disorders	10 (0.084)	9 (0.077)	9 (0.097)

Third, we additionally compared the novelty of the technologies that cover by the gender of the inventors in terms of scope. The novelty in scope indicates the extent to which the invention expands the boundaries of technical scope and can be quantified by z-score which evaluates the vagueness of IPC combination [28, 39–41]. The Z-score quantifies the degree of the novelty as shown:

$$z_{\alpha \beta }=\frac{o_{\alpha \beta }–\mu_{\alpha \beta }}{\sigma_{\alpha \beta }}$$

where α and β are different IPC six-digit codes; *o*_*αβ*_ is the number of patents that have both α and β; μ_αβ_ and σ_αβ_ are the expected co-occurrences of α and β and its standard deviation, generated from a null model of the data that randomizes IPC arrangement while preserving IPC usage and number of patents within the data. In general, the high Z-score value represents a popular IPC combination, while low Z-score value is the novel combination of IPC. Since there can be more than one IPC combination in a single patent, the min and median values of the Z-score are used [42]. While the median value measures the degree to which the main body of a patent conforms to technological conventionality, min value measures the extent to which the invention contains a novel IPC combination.

Before calculating the z-score of cosmetic patents, we removed the IPC code: A61K 8 assigned to about 98% of patents. When only a single IPC is assigned to a patent, we consider it as missing value, not creating any novel recombination [40]. As shown in Table 4, the average *Z*_*median*_ for inventions of male, female, and mixed were 7.19, 6.91, and 10.11, respectively. The value of technological conventionality becomes greater when inventors of different genders interact in a patent. This shows that an inventor group of mixed gender generally includes highly conventional IPC combinations in their inventions compared to the groups of a single gender. On the other hand, the average *Z*_*min*_ for male, female, and mixed were 0, 0.98, and -0.96, respectively. The inventions of cosmetic technology with the interaction between men and women include more novel IPC combinations on average. These results indicate that the inventions from mixed gender group tend to have both novel IPC combinations and conventional IPC combinations, that resulting inventions can provide groundbreaking or practical solutions for specific needs. Overall, in perspectives of novelty, the average number of backward citations is less in female inventions as shown in Table 2, whereas the novelty in scope is greater in male or mixed gender group. This shows that the technical features of inventions from the female inventor groups are likely to be more innovative, having fewer existing prior arts, whereas their scope of technologies is comparatively conventional. Conversely, the inventions of the male group tend to be novel in technological scope, expanding the boundaries of the technological field, albeit with less emphasis on specific technical features that are genuinely new.

**Table 4:** Conventionality and novelty of inventions by gender.

Gender	Conventionality					Novelty				
	Average	STD	Q1	Q2	Q3	Average	STD	Q1	Q2	Q3
**Male**	7.194	7.757	2.510	5.145	10.046	0.001	9.870	-3.183	-0.328	3.344
**Female**	6.908	7.166	2.362	5.665	10.665	0.982	9.631	-2.955	0.225	5.416
**Mixed**	10.111	10.670	3.306	7.677	15.469	-0.963	14.667	-7.006	-0.917	4.847

Specifically, Table 5 presents the ratio of inventions by innovation type and gender in cosmetics technology. We define the inventions as four types based on the degree of conventionality and novelty: high conventionality and high novelty (H-H), high conventionality and low novelty (H-L), low conventionality and high novelty (L-H), and low conventionality and low novelty (L-L) inventions. Here, the level of high and low is a relatively compared to the average of the values of all patents in cosmetics technological field. H-H invention is more conventional patent than other patents in terms of technical scope, which means IPC combinations in the patent are frequently occurring in general, and also may include an element that is more novel, that is, rarely occurring IPC combination in the field. H-L invention is composed of conventional IPC combinations in general, but the no combination that is more novel than average. L-H invention has more than one IPC combinations that are more novel but the IPC combinations in general are less conventional. Most of the elements in L-L invention are neither too novel nor too conventional in their technological scope. The table shows that male inventors usually create the inventions that have high novelty in scope in general (L-H inventions) compared to those of other groups, which can result in radical innovation with high invention value. The invention value indicates the extent to which the patent receives forward citations that is highly correlated to the patent’s actually achieved value [41]. On the other hand, group of mixed gender often invent patents with more conventional IPC combinations than the groups of single gender, some with novel combinations included (H-H invention) and some with less novel combinations (H-L invention). These inventions may have relatively low inventive value regardless of novelty level since it would be difficult for novel knowledge to be the integrated into highly conventional knowledge [41]. Meanwhile, female inventors are more likely to participate in L-L invention that can bring gradual and modest improvements on existing solutions.

**Table 5:** Ratio of inventions by invention type and gender.

Gender	H-H invention	H-L invention	L-H invention	L-L invention
**Male**	0.077	0.245	**0.427**	0.251
**Female**	0.069	0.280	0.386	**0.266**
**Mixed**	**0.156**	**0.321**	0.401	0.123

Moreover, we used the text information of patents to compare the differences in the cosmetics technology characteristics by gender. We extracted an abstract that briefly summarizes the technology of the patent. We leveraged only one patent from each family patent to avoid using duplicated information. If different patents have the same family ID, the patents represent the same invention, so we retained patents with more claims. If the number of claims is also the same, we retained patents with more forward citations. We changed the uppercase to lowercase, performed stopwords, and removed words that have little contribution in constructing the topic as preprocessing of the text.

**Fig 6:**
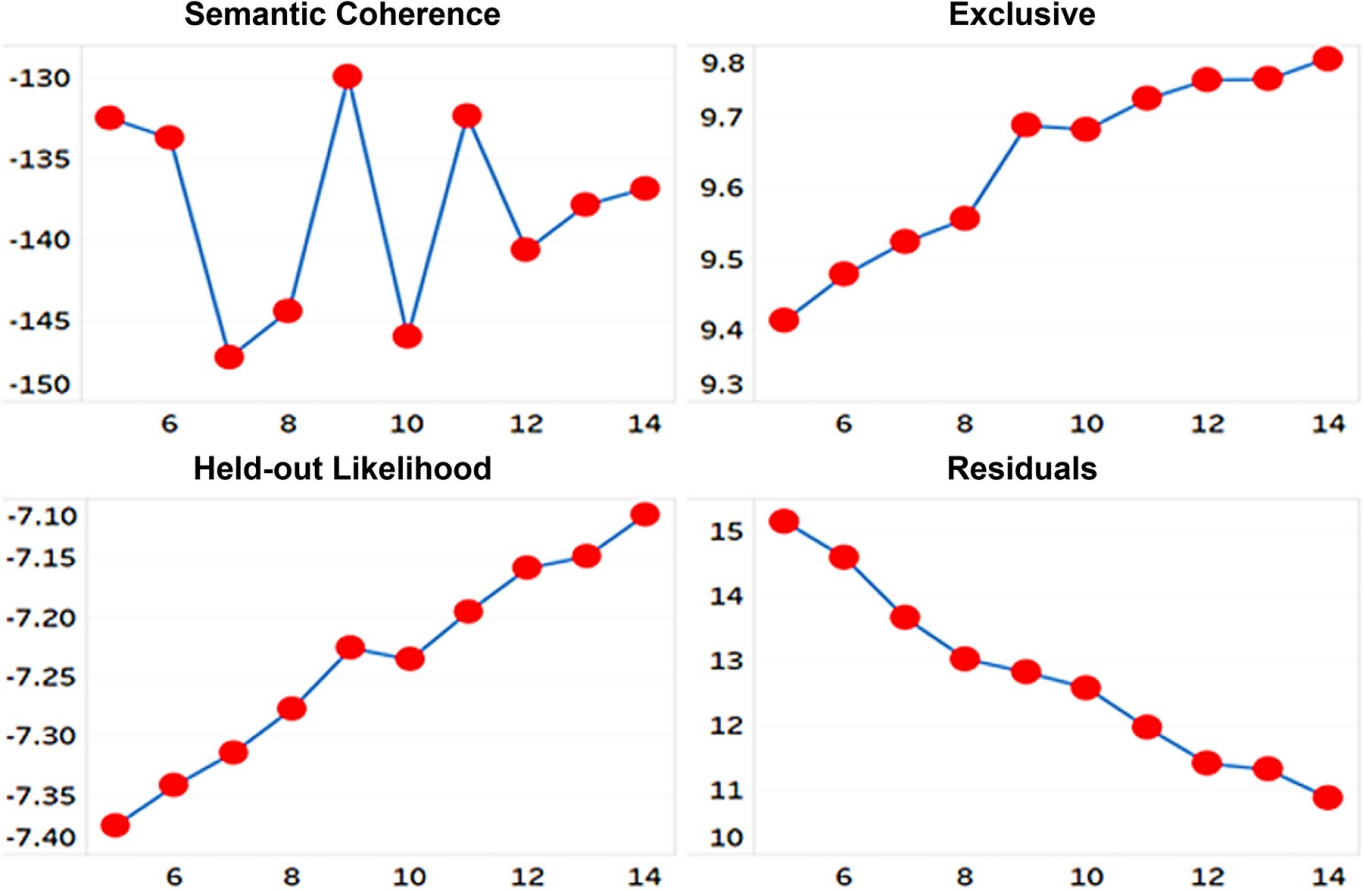
Four indicators for determining the number of topics in STM.

We conducted the structural topic modeling (STM) to observe differences in technology topics of cosmetics patents by gender. The gender of patent inventors was used as covariates. We used spectral initialization method, which conducts a spectral decomposition of the word co-occurrence matrix. This method is most often used among STM initialization methods because it shows a deterministic and globally consistent result under reasonable conditions. STM needs to pre-determine the number of topics in advance as a hyperparameter. We used four indicators to find the optimal number of topics. Semantic coherence [43] and exclusivity are indicators for evaluating the quality of the topic. Held-out likelihood and residuals are indicators for evaluating the generalization of the topic model. Through the four indicators, the optimal number of topics was 9 and 11 with the highest semantic coherence, as shown in Fig 6. We set 11 topics considering the other three indicators.

**Fig 7:**
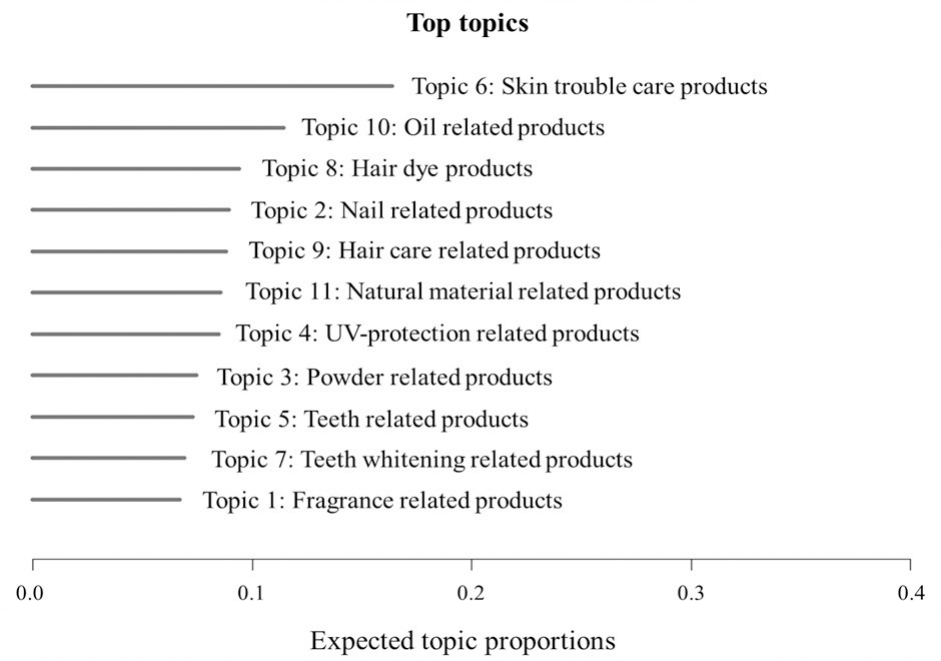
Proportion by topics.

We constructed each topic with high scored words. We calculated word score by giving higher weight to words that appear less frequently in other topics. Score divides the log frequency of the word in the topic by the log frequency of the word in other topics. The word composition of the eleven topics is shown in Table 6. We assigned the patent to one topic with the highest probability of being found among the 11 topics. Overall, the proportion of topic 6 (skin trouble care products) was the highest, as shown in Fig 7. This, again, shows that the cosmetics regarding skin trouble care have gained wide attention by inventors regardless of gender. On the other hand, topics 1, 7, 5, 3 are observed in relatively small proportions.

**Table 6:** Topics found in cosmetic patents.

No.	Words	Naming
**1**	fragrance, care, perfume, personal, odor, perfuming, flavour	Fragrance Related Products
**2**	water, nail, gel, solvent, dentifrice, deg, soluble	Nail Related Products
**3**	powder, coating, titanium, pigments, coated, color, resin	Powder Related Products
**4**	sunscreen, uv, radiation, radicals, samean, ultraviolet, hydroxyalkyl	UV Protection Products
**5**	oral, dental, teeth, tooth, fluoride, plaque, dhea	Teeth Related Products
**6**	skin, topical, cells, disorders, wrinkles, diseases, acne	Skin trouble care Products
**7**	whitening, sheet, applicator, tooth, peroxide, teeth, po4	Teeth Whitening Related Products
**8**	dyeing, keratin, fibers, dye, monomer, copolymer, fibres	Hair dye Products
**9**	hair, conditioning, surfactant, cleansing, cationic, anionic, shampoo	Hair care related Products
**10**	oil, emulsion, silicone, antiperspirant, surfactant, emulsifier, oil-water	Oil related Products
**11**	food, protein, lipid, chemical, pharmaceutical, plant, debyes	Natural Material Related Products

**Fig 8:**
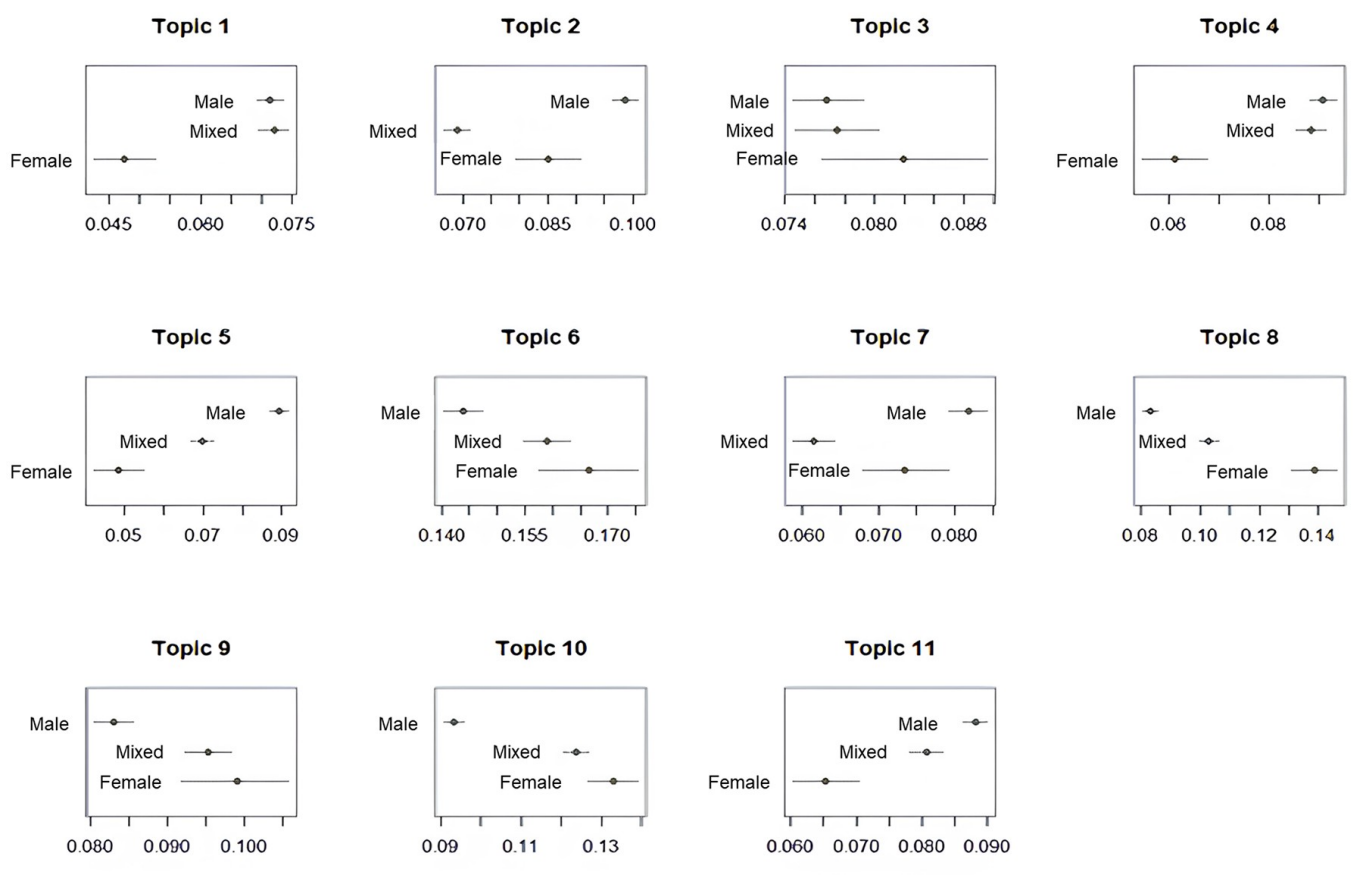
Individual topic proportion by gender.

With the powerful features of STM, we can estimate how much each covariate is associated for each topic, as shown in Fig 8. In topics 3, 6, 8, 9 and 10, it is observed that more proportion of patents is expected for those involving female inventors only. We can conclude that female inventors are more likely to invent the technologies related to comprehensive skincare, haircare, and makeup, which is widely used for female compared to male consumers due to different level of understanding on various skin types by gender and the traditional perception that improving beauty is feminine [44]. On the other hand, the proportions of topics 2, 4, 5, 7, and 11 decrease for patents with women participation. This shows that the participation of male inventors is with the inventions of well-being and personal care, which has greater demand than other cosmetic products by men customers in cosmetics market [45]. Especially, the collaborations between different genders had the lowest proportions in topics 2 and 7. Interestingly, the inventor group of mixed gender has highest proportion only in the topic 1. These trends in beauty-related topics may indicate the different interests in beauty products between genders, which may result in less collaborations between male and female inventors in patenting activities.

**Fig 9:**
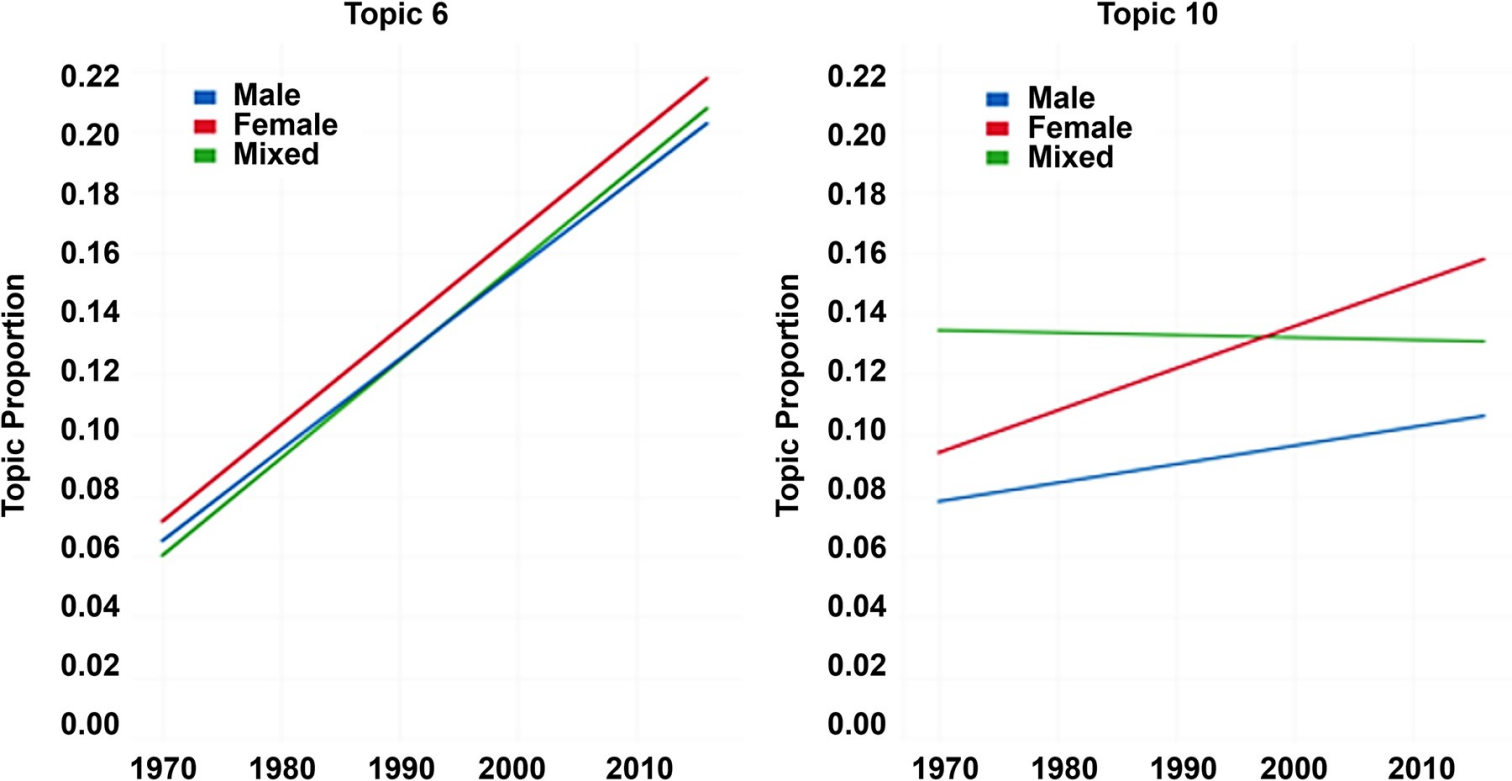
Topic proportion by gender over period on each topic by gender and year.

We estimated the prevalence for the topics, which had highest proportions, using the linear regression according to the interaction between gender and year, as shown in Fig 9. Over time, there was a significant increase in Topic 6, regardless of gender of inventors. In Topic 10, the proportion of patents with female shows a marked increase over those with other gender compositions.

### The performance of cosmetics patents

First, the most widely used indicator of performance evaluation of a patent is forward citation, which refers to the citations received from newer patents. The patents that have greater forward citations have the technological importance for the evolution of subsequent inventions [28, 29]. Hence, the number of forward citations has been used as a measure of inventive quality in previous studies [34, 46, 47].

**Fig 10:**
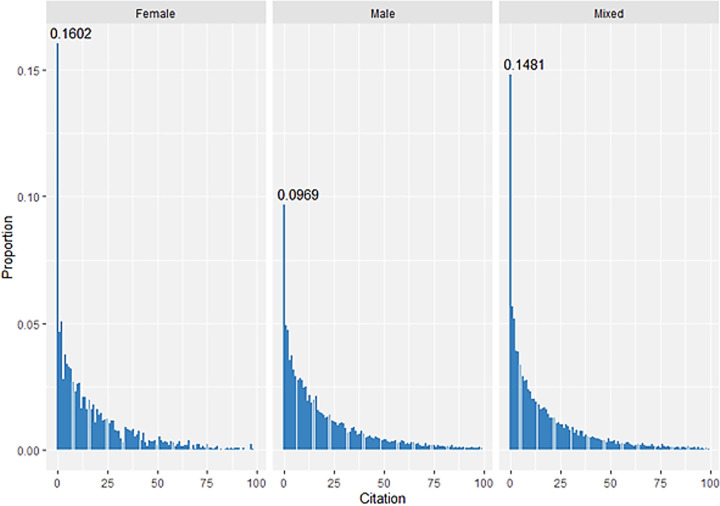
The distribution of citation by gender.

We observed the number of patents from 0 to 100 cited by gender, as shown in Fig 10. The number of forward citations for patents follows the zero-inflated Poisson distribution, which is mostly concentrated in the value of zero. More patent with female inventors were less cited than those with male.

Each patent was assigned to the topic with highest probability among 11 topics that it can have. 932 patents without abstract information in the PATSTAT database were assigned topics based on their IPC codes. The applicant affiliation consists of company, hospital, individual, non-profit organization, university, and unknown.

We conducted the zero-inflated Poisson regression to observe how gender differences affect the number of patent forward citation predictions. Of the total patents, 4% of patents with more than 100 citations were considered as outliers and were excluded from our analysis. Variables for predicting the number of forward citations for a patent include categorical variables, which are patent inventor’s gender (base level: female), patent’s topic (base level: topic 1), and affiliation inclusion (base level: no applicants assigned to each affiliation), and continuous variables, which are the number of claims, family size, the number of backward citations, the length of grant lag, the number of inventors, and the number of assigned 8-digit IPC. The number of inventors is related to the solid knowledge foundation [28, 29], whereas the number of IPCs represents the technology scope, which indicates how diversified or broad technical features a patent composed of [25, 28, 29]. These variables are proposed to have a relationship with the patent value, which is approximated by forward citation in this study. We also used the age of the patent as an offset variable to reflect the characteristic that the number of citations increased over time. The zero-inflated Poisson distribution is determined by two parameters *λ* and *π* as follows:

$$Pr(y_{j}=0)=\pi +(1-\pi )e^{-\lambda },\;Pr(y_{j}=h_{i})=(1-\pi )\frac{\lambda^{h_{i}}e^{-\lambda }}{h_{i}!}\;h_{i}\geq 1$$

Forward citation has a value of zero with the probability of *π*, and follows a Poisson distribution with a probability of 1−*π* having a parameter of *λ*. Parameters *λ* and *π* are estimated as follows:

$$ln(\lambda )=\alpha +\beta X_{i}$$

$$logit(\pi )=ln(\frac{\pi }{1-\pi })=\alpha +\beta X_{i}$$

Table 7 shows the exponential values and p-value of the parameters *λ* and *π*. The significant variables for parameter π is the gender of inventor, which affiliation the patent applicant belongs to, and which topic the patent is allocated to. The variable that shows significant interaction with parameter *λ* most is which topic the patent is allocated to, and there is little gender difference. Inventor groups with male-only and mixed gender have positive relationships with the receiving of at least one forward citation compared to the female-only group. However, the parameter *π* of the patents of mixed gender was not statistically significant. For male, the *λ* was 0.945 times of that of female, which indicates the inventions of males get slightly fewer forward citations compared to those of females, whereas the *λ* for mixed gender group was 1.052 times of that of female. These indicate that the probability of not being cited is significantly lower in male than in female, despite the fact that the number of forward citations of already cited patents is lower than that of female inventors’ patents. Meanwhile, topics from 2 to 11 have higher probability of being cited than topic 1 (Fragrance Related Products). Moreover, they have a positive relationship with the received number of forward citations, except for topic 4 (UV Protection Products).

**Table 7:** The exponential values and p-value of the coefficients.

Parameter	*π*/1−*π*	p-value		*λ*	p-value	
**Intercept**	0.115	< 2e-16	***	0.707	< 2e-16	***
**Gender (Male)**	0.628	1.91e-08	***	0.945	< 2e-16	***
**Gender (Mixed)**	0.893	0.202799		1.052	6.34e-15	***
**Claim**	0.983	3.72e-14	***	1.009	< 2e-16	***
**Family size**	0.997	0.454906		1.006	< 2e-16	***
**IPC range**	0.965	1.77e-06	***	1.007	< 2e-16	***
**Backward citation**	0.997	0.004070	**	1.002	< 2e-16	***
**Inventor number**	1.020	0.187152		1.035	< 2e-16	***
**Grant lag**	0.996	0.000228	***	0.999	< 2e-16	***
**Company (yes)**	2.354	< 2e-16	***	0.945	6.18e-16	***
**Hospital (yes)**	1.998	0.212805		1.177	0.000515	***
**Individual (yes)**	3.333	< 2e-16	***	0.977	3.08e-05	***
**Non-profit (yes)**	3.091	1.36e-13	***	0.937	5.97e-06	***
**University (yes)**	2.645	3.47e-14	***	0.923	2.11e-12	***
**Unknown (yes)**	5.866	< 2e-16	***	0.800	2.23e-16	***
**Topic 2**	0.489	1.09e-08	***	1.105	< 2e-16	***
**Topic 3**	0.607	4.32e-05	***	1.449	< 2e-16	***
**Topic 4**	0.649	9.50e-05	***	0.906	< 2e-16	***
**Topic 5**	0.485	2.42e-09	***	1.273	< 2e-16	***
**Topic 6**	0.856	0.099702	.	1.283	< 2e-16	***
**Topic 7**	0.653	0.000710	***	1.643	< 2e-16	***
**Topic 8**	0.956	0.669037		1.108	< 2e-16	***
**Topic 9**	0.428	6.65e-13	***	1.210	< 2e-16	***
**Topic 10**	0.512	9.02e-10	***	1.392	< 2e-16	***
**Topic 11**	0.763	0.022903	*	1.220	< 2e-16	***

Significance: <0.001: ‘***’, <0.01: ‘**’, <0.05: ‘*’, <0.1: ‘.’, >0.1: ‘ ‘

Second, patent maintenance term can also be used as performance indicators. In general, when a patent is granted, the owner of the patent must pay maintenance costs to be legally protected. Hence, the period during which patent holders pay a fee for the patent maintenance indicates the implied value of a patent. We assume that the maximum lifetime of a patent is 20 years from application filling date when there are no specific legal events for the patent. The lifetime of a patent can be further extended through legal events such as Term Extension of Rights. In addition, the lifetime of a patent can be terminated by a legal event such as Lapse and Withdrawal and can be restored through a Reinstatement event.

**Fig 11:**
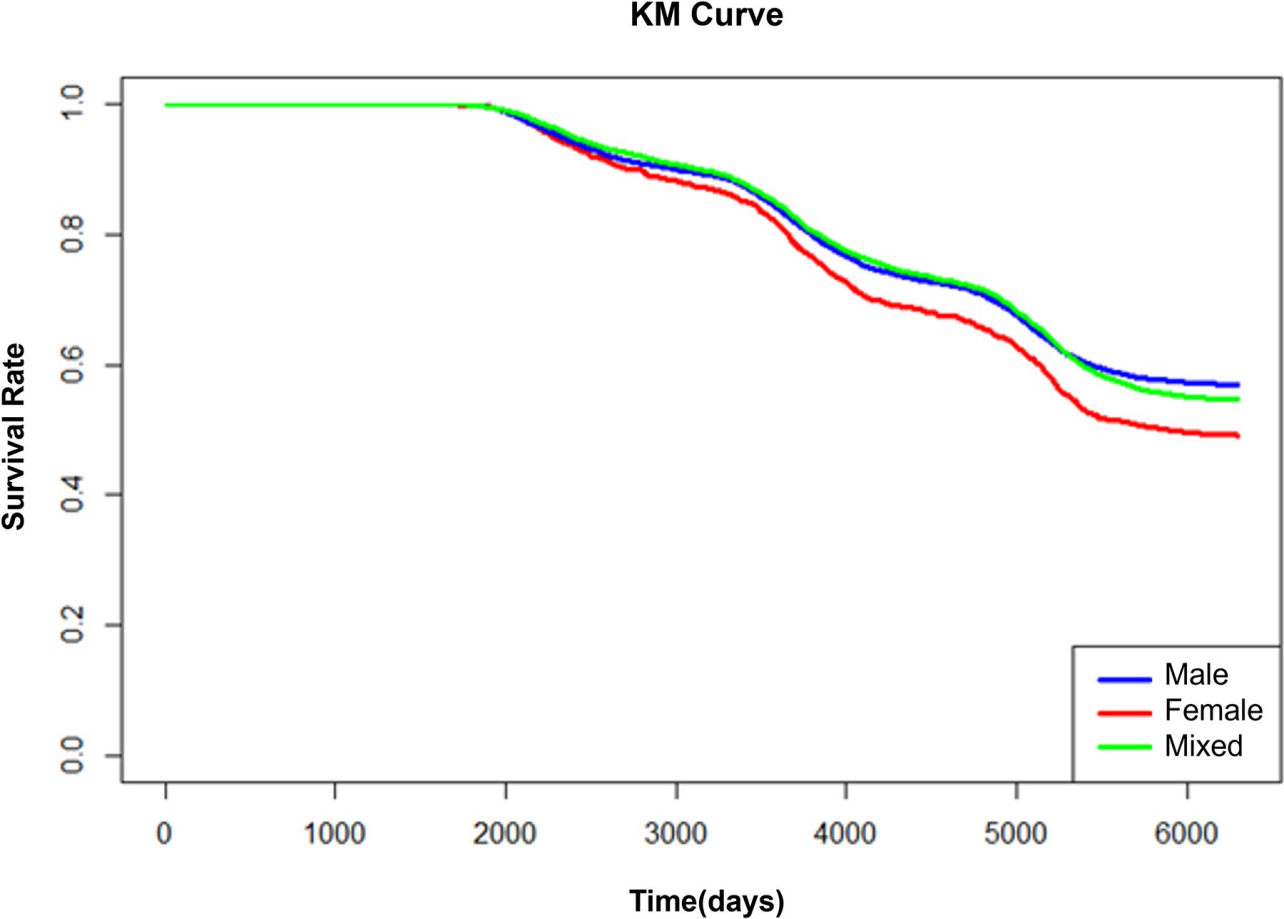
Kaplan-Meier curve of patents by gender.

The lifetime of the patent was censored as of January 1, 2018, as we used the PATSTAT’s Spring version of 2018. We conducted a survival analysis to observe if there was any difference in the lifetime of patents by gender. First, based on the data, we observed how the survival rate of patents changes according to gender through Kaplan-Meier survival analysis, as shown in Fig 11. The survival rate of those with only female participation is lower than that of male and mixed genders starting at 2000 days. In 20 years of patent maintenance term, the difference in survival rate between male and female is about 8%.

In addition, we fitted the cox proportional hazard model considering the several variables from those used in the previous analysis. The cox proportional hazard model estimates hazards, which indicate the risk that the life of a patent will be terminated at a particular point in time. The hazard function of the patent *k* at time *t* is defined as follows.


$$h_{k}(t)=h_{0}(t)e^{\beta X_{k}}$$

The result of the Cox proportional hazard model is shown in Table 8. First of all, hypothesis test regarding the proportional hazard based on Schoenfeld residuals has p-value greater than 0.05, which supports the use of the proportional hazards model. In addition, very small p-values associated with the likelihood ratio, Wald, and score tests back up the use of X variables in the proportional hazard model. The most important factor influencing the hazard of the patent was the number of claims and the composition of inventors. The hazard that the patents by male will be terminated is 0.96 times of that of female or mixed, which indicates that the patents invented by male-only teams have slightly lower risk of termination than those invented by teams of female or mixed gender. Patents with greater number of claims have a slightly higher risk of termination, but this may be different by technological industries [48]. However, there was no statistically significant difference in risk by affiliations or topics.

**Table 8:** Results of the cox proportional hazard model.

Parameter	Exp(coef)	95% Confidence limits		Z-value	P-value	
Gender (Male)	0.9553	0.9215	0.9904	-2.481	0.0131	*
Claim	1.0034	1.0022	1.0046	5.629	1.81e-08	***
Hospital (yes)	0.7746	0.3686	1.6276	-0.674	0.5001	
Non-profit (yes)	1.0842	0.9356	1.2564	1.075	0.2825	
Topic 2	0.994	0.9388	1.0523	-0.208	0.8351	
Topic 6	0.9923	0.9433	1.0439	-0.298	0.7654	
Topic 7	0.9944	0.919	1.076	-0.139	0.8894	
Topic 9	0.9801	0.9225	1.0414	-0.649	0.5166	
Topic 10	0.9799	0.9254	1.0375	-0.698	0.485	
Topic 11	1.0217	0.9463	1.1031	0.549	0.5832	
**Test**			**Value**	**DF**	**P-value**	
Likelihood Ratio Test			40.82	10	1e-05	***
Wald Test			43.46	10	4e-06	***
Score Test			43.07	10	5e-06	***
Proportional Hazards Assumption Test			17.857	10	0.0574	

Significance: <0.001 = ‘***’, <0.01 = ‘**’, <0.05 = ‘*’, >0.05 ‘ ‘

## Discussion and conclusion

### Discussion

With the advent of the modern era, as gender discrimination has been greatly resolved, women’s participation in the STEM field is rapidly increasing. In particular, women inventors have participated more in the chemical and health areas [4, 5], which are highly relevant to cosmetics technology. However, existing research on cosmetics hardly investigated the technological development regarding the gender of the inventor group. To understand the phenomena caused by these changes, this study identifies the contribution made by female expressed in cosmetic patents in terms of the three research questions.

With regard to the first research question, we observed the difference in application and grant according to women’s participation in cosmetic patent invention. In the past, most of the cosmetic patent applications were made only by male inventors, but over time, collaboration between male and female inventors has created many cosmetic patent applications. This result is in line with previous studies that demonstrated the increasing participation of female inventors in innovation, especially related to women’s life sciences. Although women’s participation in cosmetic patent applications is increasingly active, their participation in patent grants is negatively associated in cosmetics as in other sectors [8, 17].

For the second research question, we observed the difference in technological characteristics according to women’s participation in cosmetic patent invention. The collaboration between male inventors and female inventors has a positive impact on the number of backward citations and family size in cosmetic patents. Also, in terms of patent claims, the patents with participation of female had greater number of claims than the patents of male inventors. The inventor group with gender diversity also develops technology with both conventional and novel components in terms of technical scope, which can provide groundbreaking and practical solutions. These results show the technological improvement of cosmetic patents with the participation of different genders. Additionally, we observed differences in terms of the technical field by inventor groups. Within the detailed technical field extracted using STM, we notice some variation of topic prevalence depending on women’s participation. Specifically, the differences in the proportion of topics by inventors’ gender may correspond to their interests; the make-up-related topics are more likely to be invented by female inventors, while the topics regarding dental care products are more likely to be invented by male inventors [44, 45, 49]. Skin care-related topics have the highest proportions across all topics regardless of gender, of which products meeting the preferences of diverse consumer groups. These results show that the gender composition of cosmetic patents demonstrate various changes in technical characteristics.

In relation to the third research question, we observed the difference in performance according to women’s participation in cosmetic patent invention. There was little difference in the number of citations between the male inventor’s patent and the female inventor’s patent. In addition, female inventor or mixed-gender inventor group’s patent tends to be terminated slightly earlier than a male inventor’s patent. These results show that women’s participation still has a negative relationship with patent performance.

### Conclusion

This study investigates the gender diversity that affects innovation characteristics and performance in cosmetics technology. The results of the study propose that women’s participation in cosmetic inventions is becoming active and has brought about many changes in technical characteristics, but in terms of performance, it is still sluggish.

Our research contributes to the research area of cosmetics and innovation by gender. This is one of few studies that explore the patenting activities by gender in more balanced fields with less gendered stereotypes involved. Specifically, we delve into the technological characteristics and performance in various perspectives to enhance the understanding of inventors’ gender and gender diversity affecting the development of cosmetics technology. The findings of the study provide the evidence of the thematic differences and characteristics of cosmetics technology by gender participation. The stakeholders can leverage the information to plan their R&D strategies considering the landscape and market demands to better address the various needs of different genders. Specifically, based on the proposed results, the direction of technology development and collaboration strategies can be established.

As to suggestions for future research, instead of classifying the gender characteristics to three levels (male, female, mixed gender), more detailed examinations implicating the proportion of female inventors may deepen the experiment. In addition, one may investigate the differences between the patent applications and granted patents of female inventors in depth, which provide insight into topical variation or factors that decide the quality of inventions. While this study focuses on documented inventions in cosmetic technology fields and its relationship with gender composition of inventor teams as the research field entails women as the mainstream customers, generalization on the research domain is possible. The research framework provides a baseline for extending the study to other fields of gender-oriented industries, which may contribute to better understanding the complexity of gender characteristics reshaping technology development.

## References

[1] Dasgupta N., & Stout J. G. (2014). Girls and women in science, technology, engineering, and mathematics: STEMing the tide and broadening participation in STEM careers. Policy Insights from the Behavioral and Brain Sciences, 1(1), 21–29.

[2] Bottia M. C., Stearns E., Mickelson R. A., Moller S., & Valentino L. (2015). Growing the roots of STEM majors: Female math and science high school faculty and the participation of students in STEM. Economics of Education Review, 45, 14–27.

[3] Casad B. J., Petzel Z. W., & Ingalls E. A. (2019). A model of threatening academic environments predicts women STEM majors’ self-esteem and engagement in STEM. Sex Roles, 80(7), 469–488.

[4] Milli J., Gault B., Williams-Baron E., Xia J., & Berlan M. (2016). The gender patenting gap. Washington (DC): Institute for Women’s Policy Research.

[5] Koning R., Samila S., & Ferguson J. P. (2021). Who do we invent for? Patents by women focus more on women’s health, but few women get to invent. Science, 372(6548), 1345–1348. doi: 10.1126/science.aba6990 34140388

[6] Rothwell R., Freeman C., Horlsey A., Jervis V. T. P., Robertson A. B., & Townsend J. (1974). SAPPHO updated-project SAPPHO phase II. Research Policy, 3(3), 258–291.

[7] Jervis P. (1975). Innovation and technology transfer—The roles and characteristics of individuals. IEEE Transactions on Engineering Management, (1), 19–27.

[8] Jensen K., Kovács B., & Sorenson O. (2018). Gender differences in obtaining and maintaining patent rights. Nature Biotechnology, 36(4), 307–309. doi: 10.1038/nbt.4120 29621210

[9] Dunlap S. T., & Barth J. M. (2019). Career stereotypes and identities: Implicit beliefs and major choice for college women and men in STEM and female-dominated fields. Sex Roles, 81(9), 548–560.

[10] West J. D., Jacquet J., King M. M., Correll S. J., & Bergstrom C. T. (2013). The role of gender in scholarly authorship. PloS one, 8(7), e66212. doi: 10.1371/journal.pone.0066212 23894278 PMC3718784

[11] Stojmenovska D. (2023). Gender differences in job resources and strains in authority positions. Gender & Society, 37(2), 240–267.

[12] Goyanes M., De-Marcos L., Demeter M., Toth T., & Jordá B. (2022). Editorial board interlocking across the social sciences: Modelling the geographic, gender, and institutional representation within and between six academic fields. PloS one, 17(9), e0273552. doi: 10.1371/journal.pone.0273552 36054200 PMC9439229

[13] Ecklund E. H., Lincoln A. E., & Tansey C. (2012). Gender segregation in elite academic science. Gender & Society, 26(5), 693–717.

[14] Sugimoto C. R., Ni C., West J. D., & Lariviére V. (2015). The academic advantage: Gender disparities in patenting. PloS one, 10(5), e0128000. doi: 10.1371/journal.pone.0128000 26017626 PMC4446102

[15] WIPO. (2016). Special section Measuring women’s participation in international patenting.

[16] Frietsch R., Haller I., Funken-Vrohlings M. and Grupp H. (2009). Gender-Specific Patterns in Patenting and Publishing. Research Policy 38, 590–599.

[17] Miguelez E., Toole A., Myers A., Breschi S., Ferruci E., Lissoni F., et al. (2019). Progress and Potential: A profile of women inventors on U.S. patents.

[18] Jung T., & Ejermo O. (2014). Demographic patterns and trends in patenting: Gender, age, and education of inventors. Technological Forecasting and Social Change, 86, 110–124.

[19] McMillan G. (2009). Gender differences in patenting activity: An examination of the US biotechnology industry. Scientometrics, 80(3), 683–691.

[20] Cutura J. (2021). Challenges for Women Inventors and innovators in using the IP system. World Intellectual Property Organization. Retrieved from https://www.wipo.int/edocs/mdocs/mdocs/en/wipo_ip_inn_ge_21/wipo_ip_inn_ge_21_ppt_1.pdf

[21] McDermott E. (2019, February 13). USPTO: Only 4% of patents name women-only inventors in last decade. IPWatchdog.com | Patents & Intellectual Property Law. https://ipwatchdog.com/2019/02/13/uspto-only-four-percent-patents-name-women-only-inventors-in-last-decade/id=106254/

[22] Liu W. Y., Lin C. C., Lee Y. S., & Deng D. J. (2013). On gender differences in consumer behavior for online financial transaction of cosmetics. Mathematical and Computer Modelling, 58(1–2), 238–253.

[23] Jee S. J., & Sohn S. Y. (2020). Patent-based framework for assisting entrepreneurial firms’ R&D partner selection: Leveraging their limited resources and managing the tension between learning and protection. Journal of Engineering and Technology Management, 57, 101575.

[24] Mauleón E., Daraio C., & Bordons M. (2013). Exploring gender differences in patenting in Spain. Research Evaluation, 23(1), 62–78.

[25] Danish M. S., Ranjan P., & Sharma R. (2019). Valuation of patents in emerging economies: A renewal model-based study of Indian patents. Technology Analysis & Strategic Management, 32(4), 457–473.

[26] Popp D., Juhl T., & Johnson D. K. (2004). Time in purgatory: Examining the grant lag for US patent applications. Topics in Economic Analysis & Policy, 4(1).

[27] Lee S., & Lee D. K. (2018). What is the proper way to apply the multiple comparison test?. Korean journal of anesthesiology, 71(5), 353–360. doi: 10.4097/kja.d.18.00242 30157585 PMC6193594

[28] Lee J., & Sohn S. Y. (2017). What makes the first forward citation of a patent occur earlier?. Scientometrics, 113(1), 279–298.

[29] Hu Z., Zhou X., & Lin A. (2023). Evaluation and identification of potential high-value patents in the field of integrated circuits using a multidimensional patent indicators pre-screening strategy and machine learning approaches. Journal of Informetrics, 17(2), 101406.

[30] Tong X., & Frame J. D. (1994). Measuring National Technological Performance with Patent Claims Data. Research Policy, 23, 133–141.

[31] Squicciarini M., Dernis H., & Criscuolo C. (2013). Measuring patent quality: Indicators of technological and economic value. OECD.

[32] Kuhn J. M., & Thompson N. C. (2019). How to measure and draw causal inferences with patent scope. International Journal of the Economics of Business, 26(1), 5–38.

[33] Wittfoth S. (2019). Measuring technological patent scope by semantic analysis of patent claims–an indicator for valuating patents. World Patent Information, 58, 101906.

[34] Harhoff D., Narin F., Scherer F. M., & Vopel K. (1999). Citation frequency and the value of patented inventions. Review of Economics and Statistics, 81(3), 511–515.

[35] Hwang J.-T., Kim B.-K., & Jeong E.-S. (2021). Patent value and survival of Patents. Journal of Open Innovation: Technology, Market, and Complexity, 7(2), 119.

[36] Jaffe A. B., & De Rassenfosse G. (2017). Patent citation data in social science research: Overview and best practices. Journal of the Association for Information Science and Technology, 68(6), 1360–1374.

[37] Dahlin K. B., & Behrens D. M. (2005). When is an invention really radical?: Defining and measuring technological radicalness. Research Policy, 34(5), 717–737.

[38] Lanjouw J.O., & Schankerman M. (2004). Patent Quality and Research Productivity: Measuring Innovation with Multiple Indicators. The Economic Journal, 114(495), 441–65.

[39] Uzzi B., Mukherjee S., Stringer M., & Jones B. (2013). A typical combinations and scientific impact. Science, 342(6157), 468–472. doi: 10.1126/science.1240474 24159044

[40] Verhoeven D., Bakker J., & Veugelers R. (2016). Measuring technological novelty with patent-based indicators. Research policy, 45(3), 707–723.

[41] He Y., & Luo J. (2017). Novelty, conventionality, and value of invention. In Design Computing and Cognition’16 (pp. 23–38). Springer International Publishing.

[42] Kim D., Cerigo D. B., Jeong H., & Youn H. (2016). Technological novelty profile and invention’s future impact. EPJ Data Science, 5(1), 8.

[43] Mimno D., Wallach H. M., Talley E., Leenders M., & McCallum A. (2011, July). Optimizing semantic coherence in topic models. In Proceedings of the conference on empirical methods in natural language processing (pp. 262–272). Association for Computational Linguistics.

[44] Infante V. H., Calixto L. S., & Campos P. M. (2016). Cosmetics consumption behaviour among men and women and the importance in products indication and treatment adherence. Surgical & Cosmetic Dermatology, 8(2).

[45] Souiden N., & Diagne M. (2009). Canadian and French men’s consumption of cosmetics: a comparison of their attitudes and motivations. Journal of Consumer marketing, 26(2), 97–109.

[46] Harhoff D., & Hall B. H. (2002). Intellectual property strategy in the global cosmetics industry. Ludwig-Maximilians Universitaet and UC Berkeley.

[47] Henderson R., Jaffe A. B., & Trajtenberg M. (1998). Universities as a source of commercial technology: a detailed analysis of university patenting, 1965–1988. Review of Economics and Statistics, 80(1), 119–127.

[48] Choi Y. M., & Cho D. (2018). A study on the time-dependent changes of the intensities of factors determining patent lifespan from a biological perspective. World Patent Information, 54, 1–17.

[49] Grand view research (2021). Men’s Personal Care Market Size Report, 2022–2030. https://www.grandviewresearch.com/industry-analysis/mens-personal-care-market

